# Supplementary material for: Dose-Reduced FLA-IDA in Combination with Venetoclax Is an Effective and Safe Salvage Therapy in Relapsed and Refractory Acute Myeloid Leukemia (R/R AML)
Source: Cancers (Basel). 2024 Nov 19;16(22):3872. doi: 10.3390/cancers16223872 (PMC11592574; doi:10.3390/cancers16223872)
Supplement: Supplementary file 1 [file cancers-16-03872-s001.zip › cancers-3306743-supplementary.pdf]

Supplemental Material

Dose-reduced FLA-IDA in combination with venetoclax is an effective and safe salvage therapy in relapsed and refractory acute myeloid leukemia (R/R AML)

Martin Schönrock[1 †], Piet Sonnemann[1 †], Nina Michalowski[2], Michael Heuser[3], Felicitas Thol[3], Francis A. Ayuk[4], Christine Wolschke[4], Evgeny Klyuchnikov[4], Carsten Bokemeyer[1], Walter Fiedler[1 ‡], Sophia Cichutek[1 ‡ *]

[1]Department of Oncology, Hematology and Bone Marrow Transplantation with Section Pneumology, University Medical Center Hamburg-Eppendorf, Hamburg, Germany.

[2] Hospital Pharmacy, University Medical Center Hamburg-Eppendorf, Hamburg, Germany.

[3] Department of Hematology, Hemostasis, Oncology and Stem Cell Transplantation, Hannover Medical School, Hannover, Germany.

[4] Department of Stem Cell Transplantation, University Medical Center Hamburg-Eppendorf, Hamburg, Germany.

† These authors contributed equally to this work and share first authorship

‡ These authors share last authorship

***Corresponding author:**

Sophia Cichutek, email: s.cichutek@uke.de

Table of contents:

Supplemental Table S1: AHSCT strategies and median time to AHSCT.

Allogeneic hematopoietic stem cell transplantation			
	**FLA-VIDA [n, (%)]**	**FLA-IDA [n, (%)]**	**p**
Patients proceeded to AHSCT	44 (86.3)	27 (71.1)	0.133
Responding patients	35/38 (92.1)	14/18 (77.78)	0.1949
Non-responding patients	8/12 (66.67)	13/18 (72.2)	0.7761
Median time to AHSCT from FLA-VIDA/FLA-IDA start	49, SD 16.3	48, SD 10.9	0.443
Donor type			0.316
haploidentical	9 (20.5)	1 (3.7)	
MMRD	1 (2.3)	1 (3.7)	
MMUD	5 (11.4)	4 (14.8)	
MRD	6 (13.6)	4 (14.8)	
MUD	20 (45.5)	18 (66.7)	

Suppl. Table S1: AHSCT strategies and median time to AHSCT. AHSCT: allogeneic hematopoietic stem cell transplantation, MMRD: mismatched related donor, MMUD: mismatched unrelated donor, MRD: matched-related donor, MUD: matched unrelated donor, SD: standard deviation.
